# Nurses' and Physicians' Experiences With Digital Remote Patient Monitoring—Transforming the Boundaries of Breast Cancer Care

**DOI:** 10.1111/jocn.70394

**Published:** 2026-06-13

**Authors:** Maren‐Kathrine Guriby Spillum, Guro Kristine Kleivi Sahlberg, Helle Kristine Skjerven, Nina Helen MJØSUND

**Affiliations:** ^1^ Section for Breast and Endocrine Surgery, Department of Surgery, Drammen Hospital Vestre Viken Hospital Trust Drammen Norway; ^2^ Institute of Clinical Medicine, Faculty of Medicine University of Oslo Oslo Norway; ^3^ Department of Research and Innovation Vestre Viken Hospital Trust Drammen Norway; ^4^ Section for Breast and Endocrine Surgery Oslo University Hospital Oslo Norway; ^5^ Department of Mental Health Research and Development Division of Mental Health and Addiction, Vestre Viken Hospital Trust Drammen Norway

**Keywords:** breast cancer, clinical workflow, digital care, healthcare professionals, nursing, remote patient monitoring

## Abstract

**Aim:**

To explore expectations and experiences of nurses and physicians with remote care monitoring for breast cancer patients within the Norwegian specialist health service.

**Design:**

Qualitative exploratory study.

**Methods:**

Individual semi‐structured interviews were conducted with nine nurses and physicians before and after the implementation of remote patient monitoring. The data were analysed using reflexive thematic analysis.

**Results:**

Three key themes were developed: (1) ‘Navigating patient empowerment: Reassurance, misinterpretation and guidance in remote patient monitoring communication’; (2) ‘Digital care impacts the workflow: Efficiency gains and hidden burdens’; and (3) ‘Clinical judgement in a digital context: Balancing standardisation and clinical discretion’.

**Conclusion:**

While remote patient monitoring increased flexibility and targeted follow‐up, it also reshaped roles and workloads and introduced new interpretive demands that often lack formal delegation, highlighting the need for clearer task allocation and organisational support.

**Implications for the Profession:**

Remote patient monitoring expands nurses' roles in symptom assessment and digital follow‐up. As such, clear role boundaries and support for clinical judgement are essential for its successful implementation.

**Impact:**

The results are relevant for management in healthcare services, nurses and other healthcare professionals implementing remote patient monitoring.

**Reporting Method:**

The study followed CORQ guidelines.

**Patient or Public Contribution:**

Four user representatives with lived experience of breast cancer contributed to the design of the study and gave input regarding the interview guide.

## Abstract (highlights)

**What Does This Paper Contribute to the Wider Global Clinical Community?:**

- Shows how remote patient monitoring reshapes healthcare professionals' daily tasks, functions and responsibilities in oncology care.
- Demonstrates that successful digital integration depends on alignment with clinical workflows, clear roles and organisational support, not the characteristics of the technology alone.
- Provides insights to guide the development of sustainable hybrid care models that support both efficiency and professional practice.

**Highlights:**

- Healthcare professionals experienced remote patient monitoring as both beneficial and challenging in breast cancer follow‐up.
- Digital follow‐up increased flexibility and enabled more targeted patient monitoring but also introduced hidden workload and new responsibilities.
- Nurses assumed expanded roles in symptom assessment, triage and digital communication.
- Standardised symptom reporting supported clinical decision‐making but continued to require professional interpretation.
- Organisational integration, clear role boundaries and support for clinical judgement are essential for sustainable implementation.

## 1 Introduction

Remote patient monitoring (RPM) refers to healthcare practice supported by pre‐scheduled self‐reported health measurements transmitted to healthcare services through digital mobile devices (Odendaal et al. 2020). Several RPM models also enable digital patient communication with healthcare professionals (HCPs) (Bärlund et al. 2022). According to Basch et al. (2023), RPM may include functionalities for patients to send real‐time symptom alerts to their physicians and for enacting structured follow‐up, thus enhancing symptom control and supporting clinical decision‐making. The anticipated shortage of HCPs in future, combined with the increasing patient population, represents a pressing challenge that must be addressed both globally and specifically in Norway (Ministry of Education and Research 2019; World Health Organization 2022). In response to this issue, the use of RPM is increasing in Norwegian healthcare, driven by the health policy goals of enhancing efficiency, patient‐centredness and sustainability. However, the implementation of RPM brings substantial shifts in professional function, clinical workflows and technological competence that require careful attention in order to ensure its long‐term sustainability (Aanes et al. 2024; Basch et al. 2023; Konttila et al. 2019; Sollie et al. 2023).

## 2 Background

Breast cancer is the most prevalent type of cancer among women in Norway, with 4215 women diagnosed in 2024, and its incidence rate is increasing. However, as a result of the national screening programme and advances in treatment, the 5‐year overall survival rate has reached 92.8% (Cancer Registry of Norway 2025). For patients with large tumours, extensive lymph node involvement or aggressive disease types, systemic chemotherapy remains an essential treatment. Problematically, this treatment is accompanied by a range of short‐ and long‐term side effects, such as nausea, neuropathy, postmenopausal symptoms, altered sexuality and persistent fatigue, all of which can substantially impair quality of life (QoL) (NBCG 2025; Runowicz et al. 2016). As such, monitoring of chemotherapy side effects is essential to ensure the management of adverse events and, ultimately, to support the overall well‐being and QoL of breast cancer patients (Basch et al. 2023). As a result of the increasing number of patients, oncological outpatient services are under significant pressure to uphold high‐quality patient care and maintain clinical satisfaction (Sivanandan et al. 2021).

In this context, RPM is increasingly being used in cancer care as a strategy to improve QoL, patient satisfaction and follow‐up efficiency. Studies suggest that RPM may reduce the need for in‐person consultations, allowing patients to spend less time in hospital (Sivanandan et al. 2021), whilst also supporting symptom monitoring and strengthening safety and engagement, especially when combined with electronic patient‐reported outcomes measurements (ePROMs) (Kotronoulas et al. 2014; Suchodolska and Senkus 2022). ePROMs allow clinicians to identify side effects, intervene earlier and gain a more systematic overview of a patient's condition (Benze et al. 2017; Di Maio et al. 2022). Moreover, asynchronous communication tools in RPM, such as chat functions, can facilitate efficient contact and help avoid hospital visits (Bärlund et al. 2022).

Despite these benefits of RPM, HCPs have expressed concerns regarding its use (Dretzke et al. 2023; Pappot et al. 2021) in relation to increased workloads, technical barriers and uncertainty about how digital systems affect clinical workflows (Oelschlägel et al. 2021). Workflows in healthcare operate at multiple levels, including across organisations, within organisations and at the individual cognitive level, whereby professionals process and manage their own tasks (Karsh 2009). RPM may disrupt the established routines at each of these levels, meaning adjustments to functions, processes and decision‐making may be required (Dretzke et al. 2023; Oelschlägel et al. 2021).

Furthermore, the introduction of RPM represents a cultural shift that challenges traditional models of care and the caregiver–patient relationship (Meskó et al. 2017). As such, the successful implementation of RPM depends on both HCPs' digital competence and organisational support. However, variation in skill levels across and within professions is well documented, and insufficient training can hinder the integration of new technological processes into workflows (Konttila et al. 2019). Moreover, HCPs are often not adequately involved in the development of digital tools, even though their input is crucial for ensuring their practical relevance and sustainability (Greenhalgh et al. 2017).

The Norwegian Ministry of Digitalisation and Public Governance (2024) stated that Norway aims to become the most digitalised country in the world by 2030. Indeed, in 2022, Vestre Viken Hospital Trust established Norway's first virtual hospital, which included RPM for several patient groups. Subsequently, in 2023, RPM was implemented for breast cancer patients receiving chemotherapy at Vestre Viken Hospital Trust. The implementation process involved several workshops and training sessions with HCPs, and RPM was initiated and anchored at the leadership level of the hospital, reflecting a top‐down commitment to digital innovation. The process of RPM integration was initiated several months before implementation with patients and supported by clinical project leadership, a process facilitator and the digital system provider. In addition, one ‘super‐user’, an experienced oncology nurse with training in the RPM system, was appointed to support colleagues, troubleshoot and facilitate RPM's implementation in clinical practice. Overall, this preparatory phase aimed to ensure both staff familiarity with the system and alignment between the digital solution and the established clinical routines.

For breast cancer patients at Vestre Viken Hospital Trust, standard treatment involves eight outpatient consultations during chemotherapy. With RPM, this number is reduced to four in‐person visits for patients whose treatment proceeds without complications. Additionally, patients complete 14 ePROMs based on the Edmonton Symptom Assessment System (ESAS‐R), in which symptoms are rated on a 0–10 scale throughout treatment. The ePROMs focus on common side effects of chemotherapy, such as nausea, vomiting, diarrhoea, constipation, peripheral neuropathy, fatigue, sleep disturbances, mucositis and emotional distress (Basch et al. 2023; Runowicz et al. 2016). Patients' symptom ratings are colour‐coded based on the traffic light principle, which signals the urgency of follow‐up and guides HCPs in prioritising their care (Figure 1). A *green light* indicates that the treatment can proceed as planned without HCP interference, a *yellow light* signifies a cautionary state where the HCP must contact the patient for further information, and a *red light* is interpreted as a critical state that requires immediate attention from the HCP. In addition, the system includes a chat function that enables patients to communicate asynchronously with clinicians when required.

**FIGURE 1:**
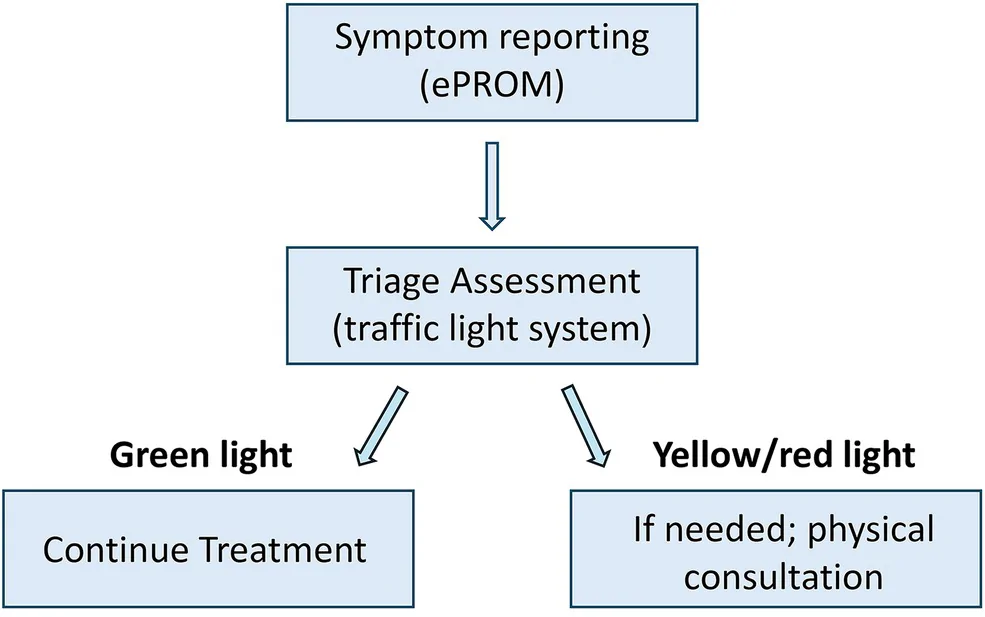
Flow chart of using RPM with patients undergoing chemotherapy. [Colour figure can be viewed at wileyonlinelibrary.com]

At present, few studies have examined how RPM is perceived and utilised by frontline HCPs in routine breast cancer care. This study aims to address this gap and, thus, generate knowledge that can help users of RPM to improve implementation and development processes and ensure that digital innovations align more closely with the realities and demands of clinical oncology practice.

## 3 The Study

### 3.1 Aim and Objectives

In this study, we aimed to explore HCPs' expectations and experiences of using RPM for breast cancer patients during the early phase of implementation in the Norwegian specialist health service. We also examined HCPs' perceptions of how RPM might influence patient–provider relationships, clinical workflows and professional roles.

## 4 Method

The study was grounded in a hermeneutic‐interpretative methodology, informed by philosophical hermeneutics as articulated by Gadamer (2006). Within this perspective, knowledge is co‐constructed through dialogue and interaction, and the interpretation of the findings is situated within the historical and social contexts of both the researchers and participants.

### 4.1 Design

In line with the hermeneutic‐interpretative methodology, the research design focused on open‐ended, dialogical data generation, which enables participants to elaborate on their own perspectives. This approach was chosen to support an in‐depth exploration of the participants' perspectives and to align with the hermeneutic emphasis on meaning‐making in context. The analysis followed an iterative interpretive process involving continuous movement between individual parts and the whole within the participants' narratives. Specifically, this process involved moving reflexively between individual statements, themes and the broader interpretative understanding of the material. Furthermore, throughout this process, the researchers' pre‐understanding and positionalities were actively reflected upon and treated as integral to the interpretations, rather than as a bias to be eliminated (Gadamer 2006). With this design, we sought to generate nuanced knowledge from individuals directly affected by the implementation of RPM. To enhance transparency and rigour in reporting, the study adhered to the Consolidated Criteria for Reporting Qualitative Research (COREQ) checklist (Tong et al. 2007). The COREQ‐checklist is provided in Appendix S1.

### 4.2 Theoretical Framework

The study framework was based on workflow theory (Alter 2013; Greenhalgh et al. 2017; Karsh 2009) and professional practice theory (Mol 2008; Molander et al. 2012; Schön 2017). Specifically, workflow theory conceptualises clinical work at three levels:

- Inter‐organisational (i.e., the coordination of tasks and information across institutions)
- Organisational (i.e., the distribution of tasks and routines within departments)
- Individual (i.e., clinicians' cognitive and practical organisation of their daily work)

Professional practice theory addresses how digitalisation shapes the following:

- Professional identity (i.e., an individual's role and responsibilities)
- Professional judgement (i.e., decision‐making based on RPM data)
- Care and responsibility (i.e., how ethical and relational considerations are maintained in a digital setting)

### 4.3 Study Setting and Recruitment

Vestre Viken Hospital Trust comprises four somatic hospitals and one division of mental health and addiction in the south‐eastern region in Norway. Prior to the study commencing, the oncology outpatient clinic at Baerum Hospital, which serves approximately 170,000 inhabitants, was preparing to implement RPM as part of breast cancer follow‐up. The RPM platform used in the clinic allowed patients to report symptoms and communicate with HCPs (i.e., nurses and doctors) through a digital platform accessible via their personal devices, such as smartphones, tablets or computers. For this, the system included a functionality for structured symptom reporting and a messaging function with HCPs that was available during defined hours.

HCPs at Baerum Hospital were eligible for inclusion in the study and were recruited in May 2023 using purposive sampling. HCP recruitment was facilitated by the department leader, who informed the staff about the study during department meetings and distributed written invitations to potential participants. HCPs who expressed interest were contacted by the first author and informed about the purpose of the study. A total of 12 healthcare professionals were eligible for participation; all were invited, and nine agreed to participate in the study. The HCPs worked with patients diagnosed with breast cancer in the curative stage of the disease, primarily in the neo‐adjuvant and adjuvant treatment pathways.

The sample size was guided by the concept of information power, which suggests that a more limited number of participants are needed when the study aim is narrow, participants are information‐rich and the analytic approach is in‐depth (Braun and Clarke 2022; Malterud et al. 2016). In our view, the research data from the interviews provided sufficient depth and variation to address the research aim, and no substantially new themes were developed in the later interviews.

### 4.4 Inclusion and Exclusion Criteria

Eligible participants were nurses and physicians directly involved in breast cancer care and expected to engage with the RPM programme. Those HCPs without such responsibilities were excluded.

### 4.5 Data Collection

Individual semi‐structured interviews were conducted both before and after the implementation of RPM at Baerum Hospital. The first round of interviews, prior to RPM implementation, took place in 2023 and aimed to explore the HCPs' expectations of RPM. The second round of interviews was conducted approximately 1 year after RPM implementation and focused on the HCPs' experiences of RPM in their daily working lives.

The interview guide for the interviews prior to RPM implementation was informed by previous research in the field and the theoretical framework and included open‐ended questions and prompts to encourage participants to elaborate on their experiences. The guide was developed with input from a user representative group comprising individuals with lived experience of breast cancer, and a panel of senior researchers with clinical expertise contributed to ensuring the guide's relevance, clarity and significance. Two pilot interviews were also used to further refine the guide. For the second interview following RPM implementation, a revised version of the guide was used, which was shaped by preliminary findings from the initial interviews in addition to the above input.

During the interviews, the interview guides were used flexibly rather than being followed strictly, thus enabling the interviewer to adapt the questions and follow‐up prompts according to each participant's responses. Transcripts were not returned to the participants for comment or correction. In line with reflexive thematic analysis, the aim of the data collection was not to seek validation of individual transcripts but to interpret patterns of meaning across the dataset (Braun and Clarke 2022). All the interviews were conducted in Norwegian and in an undisturbed office at the participants' workplace. The interviews were audio‐recorded and transcribed by the first author, a registered oncology nurse with several years of clinical experience in breast cancer care. The interviews lasted between 35 and 55 min. The two interview guides are presented in Appendix S2.

### 4.6 Data Analysis

We followed Braun and Clarke's six‐phase model for reflexive thematic analyses, including the following steps: (1) familiarisation with the data, (2) coding, (3) generating initial themes, (4) developing and reviewing themes, (5) refining, defining and naming themes, and (6) writing up (Braun and Clarke 2022). Reflexive thematic analysis, as described by Braun and Clarke (2022), was chosen because it provides a flexible approach for identifying patterns of meaning across participants' accounts. Although the interviews followed a semi‐structured interview guide, the analysis was conducted inductively, allowing themes to be developed through iterative and reflexive engagement with the data rather than being predetermined by the theoretical framework. The analysis proceeded via a hermeneutic spiral, iteratively moving between parts (e.g., individual statements) and the whole (e.g., broader themes or narratives), while explicitly engaging the researchers' pre‐understandings as resources for deepened insight and interpretation (Gadamer 2006).

The data were initially coded by the first author. To enhance analytical rigour, the codes and themes were discussed mainly with the last author, as well as the other co‐authors, throughout the analysis process. The analytical process began with repeated listening to the audio recordings and thorough reading of the transcripts to ensure immersion in the material. Each interview was coded independently and inductively, applying an open and flexible approach, in collaboration between the first and last authors.

Once the entire dataset had been coded, the codes were grouped and related to identify patterns of shared meaning. The interviews conducted before and after RPM implementation were analysed separately through phases 1–4, while in phase 5, the themes across the two interview rounds were compared and synthesised. The analytical process was iterative and recursive rather than linear, as it was characterised by flexibility, reflexivity and active researcher engagement (Braun and Clarke 2022). NVIVO 14 (Lumivero 2023) and a coding tree were employed as analytical tools to structure and visualise the relationships between the codes and themes, thus supporting an iterative and reflexive process of theme development.

### 4.7 Rigour and Reflexivity

Reflexivity, the ongoing process of critically examining how the researcher's assumptions and professional background may influence the findings (Braun and Clarke 2022), was embedded throughout the study. Specifically, during the interviews, the participants were approached as experts of their own experiences, and the interviewer ensured to ask for clarification rather than presupposing the participants' meanings. Additionally, the influences of various contextual factors, such as the Norwegian hospital setting and the participants' ages and levels of experience, on the participants' experiences were considered. No prior relationship was established between the interviewer and the majority of the participants before study commencement, as the interviewer worked in a different department. However, limited prior professional contact had occurred with one participant in relation to patient care, although this interaction was not ongoing at the time of the interviews. This contact was considered unlikely to influence the data collection but was acknowledged as part of the researcher's contextual position. The first author's clinical background in breast cancer care and prior involvement in RPM initiatives provided contextual sensitivity while also presenting a potential source of bias. To address this issue, after each interview, preliminary analytic impressions were documented together with reflexive notes reflecting the interviewer's pre‐understanding. Finally, to maintain research integrity, the data analysis process was conducted collaboratively with the co‐authors.

### 4.8 Ethical Considerations

The study was approved by the data protection officer at Vestre Viken Hospital Trust (reference number 23/04251‐7). The participants gave written consent for their participation. All the participants were given the opportunity to withdraw their consent and have their data deleted unless the data had already been included in the analysis. COREQ guidelines for qualitative research (Tong et al. 2007) were adhered to throughout the study. The interview audiotapes were stored and transcribed using ‘Nettskjema‐diktafon’. Nettskjema is a platform developed and provided by the University of Oslo that allows for the storing and handling of data in line with guidelines for research and GDPR (University of Oslo 2024).

## 5 Findings

### 5.1 Participant Characteristics

A total of *N* = 9 participants, including oncology nurses (*n* = 5) and oncologists (*n* = 4), were included in the study. The characteristics of the participants are presented in Table 1.

**TABLE 1:** Characteristics of the participants.

Gender	
Female	7
Male	2
Age	
Years, mean (range)	45.5 years (32–62)
Profession	
Oncologist	4
Oncology nurse	5
Length of work experience	
Years, mean (range)	11.2 years (4–25)

### 5.2 Themes

The findings are presented thematically, capturing both the participants' pre‐implementation expectations and, subsequently, their perceptions of how RPM impacted existing clinical structures, their professional judgement and patient follow‐up based on 1 year of use. The analysis identified the following three main themes:

1. Navigating patient empowerment: Reassurance, misinterpretation and guidance in RPM communication
2. Digital care impacts the workflow: Efficiency gains and hidden burdens
3. Clinical judgement in a digital context: Balancing standardisation and clinical discretion

#### 5.2.1 Navigating Patient Empowerment: Reassurance, Misinterpretation and Guidance in RPM Communication

Prior to RPM implementation, the participants highlighted its potential to strengthen patient empowerment. The participants hoped that RPM would make it easier for patients to contact HCP, reduce their feeling of being a burden to the HCP and increase control. These expectations of RPM were confirmed 1 year later. Several participants described how the chat function in the RPM created a sense of accessibility for the patients:

Several patients feel reassured having the chat available…That doesn't necessarily mean they use it, but they feel safer knowing it is there. (Participant 5)

The participants' expectations about patients having the opportunity to communicate in their own time and on their own terms using RPM were also fulfilled. One participant highlighted that the use of RPM gave patients more opportunity to reflect on their own health status:

Maybe patients reflect more at home when they fill out the forms… I think it can be an advantage that they have more time to think. (Participant 8)

Concurrently, several participants expressed concern that the chat function could lower the threshold for contact, potentially leading to unnecessary enquiries:

The threshold can become a bit too low, and we may end up spending a lot of time on unnecessary communication. (Participant 2)

After 1 year of RPM use, these concerns were confirmed. The participants observed that some patients had unclear expectations regarding the purpose and use of the chat function, leading to complex medical questions better addressed during clinical consultations. The participants also described how expectations regarding response times caused frustration among some patients, prompting them to contact HCP through multiple channels:

We've received a lot of strange requests through the chat… We need to be more precise when introducing this. We've had feedback that it takes too long to get a response in the chat, and then they make a phone call in addition. We're not constantly monitoring the chat, so we end up with double contact. (Participant 5)

The participants described a need for clear guidelines. The participants emphasised that the patients' interpretations of their symptoms could increase misleading reporting and thereby increase workload for the HCP. Prior to implementation, several participants expressed concern about patients underreporting symptoms. However, 1 year after, the participants described incidents of overreporting, with some patients reporting high symptom scores:

[On a scale from 1 to 10] They rated eight, and when I call and ask, they're maybe out walking saying, ‘Yeah, I'm just on my way to training now’… (Participant 1).

Before implementation, the participants expressed concerns that RPM could lead to a reduction in face‐to‐face contact between patients and HCPs and, thus, weaken the relationship between them:

Digitalisation can lead to less patient contact. Being face‐to‐face with a patient, that's something I believe all HCPs appreciate. You don't become an HCP if you don't like patient contact. That's unfortunate, because it's such an important part of the job. (Participant 3)

After 1 year of experience with RPM, concerns regarding face‐to‐face contact appeared more nuanced. While the participants described that some patients continued to express a desire for face‐to‐face contact, they also expressed that RPM does not necessarily reduce human closeness:

Our way of working has changed. We need to be more efficient and cannot spend as much time with each patient as before. Patients mainly come for treatment and some human contact, which we can still provide despite RPM. (Participant 6)

Overall, the participants described advantages of RPM for patient empowerment and healthcare accessibility, but drawbacks related to unclear communication and symptom reporting issues.

#### 5.2.2 Digital Care Impacts the Workflow: Efficiency Gains and Hidden Burdens

Participants described positive experiences with the RPM implementation and training process. They appreciated being involved in the planning of the RPM implementation and described the training, conducted in collaboration with the process facilitator and system vendor, as adequate. ‘Super‐users’ were regarded as essential for building confidence in using RPM, but they also highlighted the need for continuous development. After implementation, the RPM system was described as useful and future orientated. A ‘super‐user’ emerged as the main success factor, providing continuity and a vital link to the vendor.

We received good training. We've had the ‘super‐user’ with us the whole time. It wouldn't have worked if we hadn't had someone dedicated to it. (Participant 6)

After 1 year of use, the participants described RPM as a system with considerable potential for enabling flexible and accessible patient follow‐up. Concurrently, with the implementation of RPM, there was a need for awareness of its impact on the HCPs' workflows. The participants reported that RPM was not yet embedded into clinical routines, and active attention and effort were required to integrate RPM into everyday clinical practice rather than occurring seamlessly:

We work in three, four, five parallel documentation systems… and the RPM platform naturally comes in fourth place. (Participant 4)

Prior to RPM implementation, the participants expressed expectations that RPM could streamline routine follow‐up, increase HCPs' time availability and enable the management of more patients without increasing the overall use of human resources. Several participants hoped that routine consultations could be phased out and that digital reporting could be used to identify patients requiring a more intensive follow‐up. These expectations were partially confirmed. RPM enabled a reduction in outpatient appointments for patients with stable conditions, providing increased flexibility and control. In addition, simple enquiries were handled digitally, streamlining workflow and increasing resource availability:

I think you get a quick overview of the patient's situation. We avoid those phone calls that are time‐consuming because the patient often comes up with a lot of extra information we don't strictly need. (Participant 9)

The participants reported that this increased efficiency did not necessarily equate to less work but rather a shift in the type and intensity of tasks. Prior to RPM implementation, the work involved both low‐complexity and high‐complexity consultations, whereas later involved fewer patient appointments but more demanding encounters:

I have fewer routine assessments now, and that makes each consultation feel heavier. (Participant 4)

In addition to altering the number and content of consultations, the implementation of RPM contributed to a gradual redistribution of responsibilities. Tasks such as symptom assessment, blood test interpretation and treatment initiation was increasingly undertaken by nurses, often without formal role clarification. Department leaders sought to address these changes by clearly delineating responsibilities, such as by assigning one nurse to manage follow‐up through the chat function and digital forms. While this delineation was perceived as helpful, it required sufficient local capacity and coordination. Several participants further described this shift in responsibilities as causing an increased professional and ethical burden:

I feel a much greater responsibility when administering the chemotherapy treatment now. I ask the patients more questions than what's on the form, because I think, now I really need to be sure that the patient is OK. (Participant 1)

In practice, the participants reported that the workload was not reduced after RPM implementation but rather became less visible and more complex. The participants reported that issues that would previously have been addressed during scheduled consultations would arrive as fragmented messages distributed across the day and through multiple channels. Additionally, the reduction in in‐person appointments meant that more information had to be provided in the initial patient meeting, whereas previously, information provision would have been spread over time. Consequently, RPM introduced greater demands for planning, communication and sustained professional attentiveness:

There might be enquiries from patients that would have been brought up at the next appointment. It becomes extra important to say everything you need to during the first appointment, because it's such a long time until the next one. (Participant 6)

Overall, the participants described RPM as increasing efficiency and flexibility in patient follow‐up while also introducing hidden burdens that made the workload less visible and more complex.

#### 5.2.3 Clinical Judgement in a Digital Context: Balancing Standardisation and Clinical Discretion

Prior to RPM implementation, the participants expected that RPM would enable more individualised assessments. They hoped that RPM would support systematic and continuous symptom reporting, provide a better basis for clinical decisions, and reduce variation in care. In practice, many of these expectations were fulfilled, and the participants experienced that the symptom forms in the RPM system provided valuable insight and structure. The forms helped to identify symptoms not otherwise reported and served as tools for raising important topics during consultations:

It has been an advantage to receive a completed form before speaking with a patient, as it provides insight into the most relevant issues ahead of the consultation. Having such information in advance allows the consultation to be more targeted. (Participant 3)

The participants also viewed the chat function in the RPM enabled HCPs to read and reflect on a message before responding, which provided the opportunity for deliberation and quality assurance:

I can read the message and prepare before I respond. On the phone, you're somewhat caught off guard. This way, I can check with a doctor if I'm unsure how to answer… It feels more quality‐assured. (Participant 8)

Concurrently, the chat function also generated uncertainty regarding patients' subjective interpretations of their symptoms, making clinical discretion necessary to validate symptom scores. The forms often required follow‐up and further evaluation to ensure accurate interpretation:

When the patient rates themselves, it can look more serious, and that triggers double work… You must call them and ask them several follow‐up questions. (Participant 4)

The participants highlighted that such structured approaches to symptom assessment may have inherent limitations. For example, some patients perceived the predefined questions as too narrow and insufficiently representative of their situation. Although certain questions in the form included open‐text fields, the participants reported that some patients struggled to articulate their experiences in the way they intended:

The last question in the symptom form… that's where you get the open text fields. So, we've kind of solved it, but there are probably some who don't find or open the text field, or they want to express more. (Participant 5)

RPM provided structure to patient consultations and follow‐up, supported more individualised assessments and helped to expose symptoms that might otherwise have remained unidentified. Concurrently, the subjective nature of patient reports and the limits of predefined questions made clinical discretion and follow‐up essential.

## 6 Discussion

We aimed to explore HCPs' expectations and experiences related to changes in clinical workflows and patient interactions following the implementation of RPM for patients undergoing chemotherapy treatment for breast cancer. The findings demonstrate the dual role of RPM in cancer care and reveal both perceived benefits and emerging challenges, highlighting the complex interplay between the technological potential and clinical reality of using RPM systems.

Prior to the implementation of RPM, the participants expected it to reduce the number of routine check‐ups and enable more individualised care through better use of the HCPs' time and expertise, in line with Bärlund et al. (2022). However, these expectations were only partly realised. Patients with stable conditions could be safely followed up remotely using RPM, and the participants reported greater flexibility in tailoring care to their patients' needs. However, the perceived efficiency gains were not necessarily synonymous with a reduced workload for the HCPs. Instead, RPM caused task redistribution, making the workload less visible and more fragmented. For example, while RPM led to fewer patient encounters overall, the in‐person consultations that remained became more complex and emotionally demanding. These findings resonate with Wosny et al. (2023), which argued that digital restructuring in healthcare tends to concentrate complexity and contribute to increased cognitive and emotional strain for frontline staff.

The anticipated benefits of the chat communication proved equivocal. It appeared to enhance patients' engagement in their own treatment. However, it simultaneously introduced new expectations and potential uncertainties from the patients. Oelschlägel et al. (2021) argued that digital solutions such as chat functions give reassurance by signalling HCPs' availability and attentiveness. However, in this work, it generated a continuous stream of microtasks distributed across several communication platforms. Consistent with the findings of Pappot et al. (2021) and Wosny et al. (2023), the need to monitor multiple channels, often outside the structure of regular consultations, fragmented attention and contributed to increased load and invisible work. Moreover, unclear boundaries regarding the purpose and use of RPM occasionally led to misunderstandings and inefficiencies. For example, the chat function was used for complex enquiries better suited for face‐to‐face consultations, as also reported in other recent evaluations of digital health interventions (Dretzke et al. 2023). Despite these challenges, the participants expressed that RPM, especially the chat function, promoted a sense of continuity and presence for the patients, aligning with prior research showing that RPM can strengthen patient perceptions of safety and predictability (Aapro et al. 2020; Oelschlägel et al. 2021). However, these benefits appear contingent on the patients' understanding of the system and the HCPs' ability to guide its appropriate use. These issues with the RPM chat reported by the participants highlight the importance of clear and explicit communication and patient education concerning the practical use of RPM chat functions. Overall, this work suggests that, when used appropriately, RPM chat functions hold the potential to strengthen patient safety.

Aside from the issues with the chat function, the introduction of RPM placed increased demands on professional competence, coordination and communication, particularly for nurses. Interpreting symptom reports and reviewing laboratory results, which require greater use of clinical judgement, became integrated into daily routines. The nurses also described an increased sense of responsibility in assessing patients' suitability for new chemotherapy regimens in the absence of face‐to‐face medical evaluation. Similarly, Molander et al. (2012) argued that RPM extends nurses' responsibility for making accountable and well‐reasoned clinical judgements. As such, in this work, although the participants reported receiving sufficient training in the technical use of RPM, they felt inadequately prepared for its impact on their daily workflow. In line with the findings of Konttila et al. (2019), the participants described how using RPM meant that a more proactive and holistic approach was required during the early stages of treatment, when trust and understanding with the patient are established. Some participants expressed ambivalence regarding replacing in‐person consultations with RPM during this sensitive treatment phase, as they were unsure about whether RPM could capture the full scope of a patient's situation. These concerns highlight the need for critical differentiation between the various phases of the care pathway to identify where digital solutions may be appropriate and where physical presence and relational dialogue remain indispensable, as suggested by Odendaal et al. (2020) and Stavrou et al. (2022).

Karsh (2009) argued that the cognitive workflow, which refers to HCPs' understanding of what a consultation should entail, is often overlooked during the implementation of new routines in healthcare. Despite being involved in the integration of RPM, the participants in this study described that, following its implementation, consultations required more preparation, interdisciplinary collaboration and digital infrastructures. As such, we suggest that the implementation of RPM not only changes observable work tasks but also reshapes HCPs' cognitive workflow. This suggestion is in line with Greenhalgh et al. (2017), who argued that digital transformation in healthcare rarely leads to simplification and, instead, tends to reconfigure work practices, generating invisible work and requiring new types of interprofessional trust and coordination. Therefore, we argue, in line with Wosny et al. (2023), that as the use of RPM increases, new needs will arise, and clear routines, shared decision‐making frameworks, and robust systems are needed to ensure patient safety.

In line with the findings of Pappot et al. (2021) and Astrup et al. (2022), the participants in this study reported that the digital forms in the RPM platform uncovered symptoms that might otherwise not have been detected, thus enhancing diagnostic sensitivity. However, while the findings indicate that RPM offers advantages in terms of providing structure and systematisation, there are also important limitations, particularly in relation to the complexity of clinical experience. For example, the results of this work highlight the need for clinical discretion in the interpretation of symptom scores, reinforcing Di Maio et al. (2022), who advised caution against overreliance on self‐reporting tools without clinical contextualisation. In the present work, the participants frequently described how patient‐reported data required careful contextual interpretation, and follow‐up calls to clarify the meaning behind and severity of high symptom scores. These results reflect the ‘interpretive gap’ described by Suchodolska and Senkus (2022), which refers to the difference between standardised data and the nuanced judgements that constitute clinical reasoning.

In several cases, high symptom scores were not indicative of acute symptom burden but functioned as expressions of uncertainty, emotional need or a desire to be seen by HCP. Such scores do not necessarily represent misuses of the system but illustrate the diverse ways patients use digital tools to articulate complex needs, concurring with the findings of Oelschlägel et al. (2021). The findings of the present study underscore how standardised forms do not always accommodate patients' expressive needs and that important contextual information may be lost when communication is reduced to numerical categories. This loss of contextual information may weaken the basis for accurate clinical assessment and timely intervention (Di Maio et al. 2022). Overall, since RPM may not adequately meet all patient needs, HCPs must remain attentive to this limitation. Patients who rate their symptoms as high without presenting an acute problem may still require additional telephone or in‐person consultations to ensure appropriate support.

Moreover, the digital modality introduces a new temporal dynamic to patient‐HCP relationships. In this work, the chat function was perceived as positive and provided HCPs with time to reflect, consult colleagues and formulate appropriate responses, enhancing quality and being professionally rewarding. However, using the chat depended on organisational systems capable of ensuring timely and coordinated responses, particularly in high‐risk or emotionally charged situations. When these systems functioned well, RPM was seen as a clinical asset that enhanced response quality and reinforced patients' sense of being supported, consistent with the findings of Aapro et al. (2020).

Despite the promise of digitalisation, the findings revealed a paradox in the implementation of RPM in healthcare systems. While the technology is present and its potential widely acknowledged, it remains partially embedded in everyday clinical practice. Consistent with the findings of Wosny et al. (2023) and Pappot et al. (2021), the participants in this work described that when RPM was integrated outside of established documentation systems and routines, it contributed to a sense of fragmentation and cognitive overload. The participants consistently described RPM as a system operating ‘on the side’ and as a parallel channel requiring active attention and manual effort for access, monitoring and maintenance. Rather than being seamlessly integrated, RPM appeared as a ‘shadow system’ that was visible but not fully operational and was valued yet insufficiently aligned with the core structures of clinical work. One of the key barriers to the meaningful integration of RPM was the lack of interoperability with existing platforms and electronic health records.

The tension between organisational structure and clinical discretion is a central theme in this work. Symptom forms in RPM may contribute to improved consistency, prioritisation and follow‐up in care, but can also underestimate or exaggerate the clinical picture if interpreted without contextual awareness. This dilemma highlights the importance of supplementing standardised instruments for symptom reporting with open, relational and dialogue‐based communication, especially in cases where symptom scores deviate from expected trajectories or appear ambiguous.

### 6.1 Strengths and Limitations

This study provides insights into HCPs' expectations of and experiences with RPM, but several limitations must be noted. We used a small sample of nine participants from a single clinical setting, meaning a careful evaluation of the extent to which the findings may have relevance in other contexts is required. However, the in‐depth interviews yielded richly detailed accounts of the participants' expectations of and experiences with RPM. The data also reflect the early implementation phase of RPM, and perspectives may shift as the system becomes more integrated. Additionally, the study focused on nurses and physicians, excluding other stakeholders such as managers, developers, patients and patients' next of kin. In future, their perspectives should also be explored to broaden the knowledge regarding the impact of RPM. Finally, due to the use of interview‐based methods, social desirability bias cannot be ruled out. Despite these limitations, the study offers important perspectives on how frontline staff navigate digital transformations in breast cancer care, and we argue that the findings are likely to also have relevance for other areas of care.

### 6.2 Recommendations for Further Research

The findings from this study highlight several important avenues for future investigation. Clinical workflows should be clearly examined to ensure that RPM is structurally embedded into daily routines rather than functioning as a parallel system. Future studies should also examine when and for whom RPM is most appropriate, giving particular attention to the different phases of the breast cancer care pathway. Differentiating the use of digital tools across various treatment stages may help to balance care standardisation with the provision of individualised care. Finally, further research is needed to explore how digitalisation shapes clinical judgement, interprofessional collaboration and ethical decision‐making in oncology.

### 6.3 Implications for Policy and Practice

The implementation of RPM has significant implications for healthcare delivery and service organisation. The informal expansion of nurses' responsibilities with RPM highlights the need for clearly defined roles and shared frameworks for triage and decision‐making. Moreover, the integration of RPM introduces new demands on professional competence, and thus, digital health literacy must be strengthened among both providers and patients to ensure its efficient use. Technical solutions must also be aligned with organisational capacity to avoid workflow fragmentation, particularly by ensuring interoperability with electronic health records and integration into existing clinical practice. To achieve this alignment, the implementation of RPM requires strong leadership engagement and organisational support structures. Successful RPM implementation also requires participatory service design, where HCPs, process facilitators, vendors and patients co‐develop sustainable models for hybrid care. RPM implementation cannot be reduced to the technological aspects alone, and equal attention must be given to how clinical workflows will be reshaped using the technology. Workflows in healthcare operate across organisations, within institutions, and at the individual cognitive level, and the sustainable adoption of RPM depends on aligning the technology with these interdependent processes.

## 7 Conclusion

In this study, we explored HCPs' perspectives on how RPM reconfigures patient–provider relationships, influences clinical workflows and redefines professional roles, responsibilities and judgement. The findings illustrate the complex nature of the integration of RPM into cancer care. While RPM supports increased flexibility and more targeted follow‐up, it also reconfigures professional roles, redistributes the workload and introduces new interpretive demands. These changes may add responsibilities without formal delegation, thus highlighting the need for clearer task allocation and stronger organisational support. Furthermore, patient‐reported data require careful interpretation, as high symptom scores may reflect emotional needs or uncertainty rather than clinical deterioration. As such, the findings of this work confirm the importance of maintaining clinical discretion and relational dialogue alongside structured data collection. Despite substantial efforts to integrate RPM into everyday clinical practice, the participants continued to describe challenges related to workflow alignment, responsibilities and the coexistence of parallel systems. Successful digital transformation depends not only on the technology itself, but also on social and organisational integration of the system within clinical practice that aligns with workflows, responsibilities and technology.

## Author Contributions

The study was conceptualised by M.‐K.G.S. and G.K.K.S. Data curation was performed by M.‐K.G.S. Development and design of methodology was carried out by M.‐K.G.S. and N.H.M. Formal analysis was conducted by M.‐K.G.S. Both M.‐K.G.S. and N.H.M. were involved in data analysis and interpretation. M.‐K.G.S. wrote the original draft, while N.H.M., G.K.K.S. and H.K.S. reviewed and edited it. M.‐K.G.S. is an oncological nurse with long‐term experience in breast cancer care. N.H.M. has long‐standing research experience in specialised healthcare services, employing qualitative methodologies, with participants across the lifespan, from children to older adults. G.K.K.S. is head of research and innovation in Vestre Viken Hospital Trust and a professor at the Institute of Clinical Medicine at the Faculty of Medicine at the University of Oslo with extensive experience in clinical and translational cancer research. H.K.S. is the head surgeon of breast and endocrine surgery and has long‐term experience in clinical cancer research. All authors have approved the final version of the review.

## Funding

This work was supported by Vestre Viken Hospital Trust (11/2024) and Selena‐Sisters in Drammen, Norway.

## Conflicts of Interest

The authors declare no conflicts of interest.

## Supporting information


**Appendix S1:** COREQ (Consolidated Criteria for Reporting Qualitative Research) checklist.
**Appendix S2:** Semi‐structured interview guide used during data collection.

## Data Availability

The data that support the findings of this study are available on request from the corresponding author. The data are not publicly available due to privacy or ethical restrictions.
