# Supplementary material for: Nurses' and Physicians' Experiences With Digital Remote Patient Monitoring—Transforming the Boundaries of Breast Cancer Care
Source: J Clin Nurs. 2026 Jun 13;35(11):4969–79. doi: 10.1111/jocn.70394 (PMC13629272; doi:10.1111/jocn.70394)
Supplement: Supplementary file 1 [file JOCN-35-4969-s001.docx]

**Appendix S1: COREQ checklist**

| **No.** | **Item** | **Guide question/description** | **Authors comments and changes in the manuscript** |
| --- | --- | --- | --- |
| **Personal Characteristics** | | | |
| **1.** | **Interviewer/facilitator** | Which author/s conducted the interview or focus group? | The first author conduced the interviews. |
| **2.** | **Credentials** | What were the researcher's credentials? E.g. PhD, MD | The first author is a registered oncology nurse, and the co-authors hold PhD degrees. |
| **3.** | **Occupation** | What was their occupation at the time of the study? | The author’s occupation is presented under “Authors Contribution”. |
| **4.** | **Gender** | Was the researcher male or female? | All were females. |
| **5** | **Experience and training** | What experience or training did the researcher have? | The author’s experience is presented under “Authors Contribution”. |
| **Relationship with participants** | | | |
| **6.** | **Relationship established** | Was a relationship established prior to study commencement? | No prior relationship was established with the majority of the participants. The interviewer had limited prior professional contact with one participant. |
| **7.** | **Participant knowledge of the interviewer** | What did the participants know about the researcher? e.g. personal goals, reasons for doing the research | Participants were informed about the purpose of the study and the interviewer’s professional background. No prior personal relationship or additional information about the researcher was disclosed. |
| **8.** | **Interviewer characteristics** | What characteristics were reported about the interviewer/facilitator? e.g. Bias, assumptions, reasons and interests in the research topic | The interviewer’s clinical background provided contextual understanding of the participants’ work; however, it also introduced potential assumptions regarding digital follow-up and clinical practice. In line with a reflexive thematic analysis approach, these preconceptions were actively reflected upon throughout the research process. |
| **Domain 2: Study design** | | | |
| **Theoretical framework** | | | |
| **9.** | **Methodological orientation and Theory** | What methodological orientation was stated to underpin the study? e.g. grounded theory, discourse analysis, ethnography, phenomenology, content analysis | The study was grounded in a hermeneutic-interpretative methodology. |
| **Participant selection** | | | |
| **10.** | **Sampling** | How were participants selected? e.g. purposive, convenience, consecutive, snowball | Purposive sampling was used to recruit healthcare professionals with experience in the remote patient monitoring |
| **11.** | **Method of approach** | How were participants approached? e.g. face-to-face, telephone, mail, email | Participants were approached through departmental meetings and direct invitations facilitated by the department leader. |
| **12.** | **Sampel size** | How many participants were in the study? | Nine healthcare professionals participated in the study. |
| **13.** | **Non-participation** | How many people refused to participate or dropped out? Reasons? | No participants dropped out |
| **Setting** | | | |
| **14.** | **Setting of data collection** | Where was the data collected? e.g. home, clinic, workplace | The data was collected at the participants workplace. |
| **15.** | **Presence of non-participants** | Was anyone else present besides the participants and researchers? | The Interviews were conducted in an undisturbed office with no other present than the participant and the interviewer. |
| **16.** | **Description of sample** | What are the important characteristics of the sample? e.g. demographic data, date | The sample included nine healthcare professionals (4 oncologists, 5 oncology nurses), mostly female, with a mean age of 45.5 years (32–62) and 11.2 years of work experience (4–25). |
| **Data collection** | | | |
| **17.** | **Interview guide** | Were questions, prompts, guides provided by the authors? Was it pilot tested? | A semi-structured interview guide with open-ended questions and prompts was developed by the research team. The interview guide was pilot tested prior to the data collection. |
| **18.** | **Repeat interviews** | Were repeat interviews carried out? If yes, how many? | The were conducted two interviews one before end one after the implementation of remote patient monitoring. |
| **19.** | **Audio/visual recording** | Did the research use audio or visual recording to collect the data? | The interviews were audio recorded. |
| **20.** | **Field notes** | Were field notes made during and/or after the interview or focus group? | After each interview, initial themes that immediately came to the interviewer’s mind were written down, together with reflexive notes on the interviewer’s pre-understanding. |
| **21.** | **Duration** | What was the duration of the interviews or focus group? | The duration of the interviews was between 35-55 minutes. |
| **22.** | **Data saturation** | Was data saturation discussed? | Data saturation was not used; instead, sample size was guided by the concept of information power. |
| **23.** | **Transcripts returned** | Were transcripts returned to participants for comment and/or correction? | Transcripts were not returned to participants for comment or correction. |
| **Domain 3: Analysis and findings** | | | |
| **Data analysis** | | | |
| **24.** | **Number of codes** | How many data coders coded the data? | The first author initially coded the dataset. Further, the codes and teams were discussed mainly with the last author, but also with the rest of the research team. |
| **25.** | **Description of the coding tree** | Did authors provide a description of the coding tree? | A formal coding tree was not presented; however, a coding tree was used as an analytical tool to structure and visualise the relationships between codes and themes. |
| **26.** | **Derivation of the themes** | Were themes identified in advance or derived from the data? | Themes were derived inductively from the data through an iterative coding process. |
| **27** | **Software** | What software, if applicable, was used to manage the data? | We used “Nettskjema” with an accompanying Dictaphone to record and store the interviews. “nettskjema” is a safe please to collect and store data provides by the University of Oslo. In addition, we used NVIVO to manage data. |
| **28.** | **Participant checking** | Did participants provide feedback on the findings? | No. Participants did not provide feedback on the findings. |
| **Reporting** | | | |
| **29.** | **Quotations presented** | Were participant quotations presented to illustrate the themes / findings? Was each quotation identified? e.g. participant number | The participants quotations are presented with e.g. Participant 5. |
| **30.** | **Data findings consistent** | Was there consistency between the data presented and the findings? | In our view, there was consistency between the data and the findings, supported by illustrative participant quotations. |
| **31.** | **Clarity of major themes** | Were major themes clearly presented in the findings? | We consider that major themes were clearly presented in the results section and illustrated with supporting quotations. |
| **32.** | **Clarity of minor themes** | Is there a description of diverse cases or discussion of minor themes? | We assess that minor themes and variations in participants’ perspectives were described within and across the main themes. |

**Appendix S2**

**Interview 1- Expectations Healthcare Professionals**

**Background information**

- What is your role within the department?
- How many years have you worked with oncology patients?
- What are your previous experiences with digital health tools? How did they work in practice?

**Training and information**

- To what extent do you feel adequately informed about the implementation of Remote Patient Monitoring (RPM)?
- How has the supporting team facilitated training and implementation?
- If you are missing specific input:
  - What kind of training or education would you have preferred?
- In your opinion, what prerequisites must be in place prior to implementing RPM?

**Expectations regarding implementation**

- What are your expectations for RPM?
- What benefits do you anticipate RPM might bring?
- In what ways could it be useful?
  - Useful for whom (patients, healthcare professionals, management, or the healthcare system)?
- Do you foresee any disadvantages or drawbacks? What are they, and who might they affect (patients, healthcare professionals, management, or system-level)?
- Do you have any concerns ahead of RPM implementation?
  - If so, what are your concerns specifically related to?
- In your view, how might RPM affect the relationship between healthcare professionals and patients?
- Do you anticipate that your work situation will change?
  - If so, in what way?
  - If not, why not?
- Do you think RPM will affect patients’ sense of safety and being cared for?
  - In what way?
  - Why or why not?
- Can you foresee any potential challenges or issues arising during the implementation or use of RPM?

**Current clinical practice**

- How do you currently follow up with patients? What type of healthcare services are you providing?
- Why is follow-up structured this way?
- How would you assess the quality of the current care provided?
- Can you envision how aspects of the current practice might be transferred to a RPM context?
- Are you concerned that some areas of patient follow-up may be compromised with the use of RPM?
  - If so, which areas?
- How do you imagine that interaction and communication with patients will change with the use of RPM?

**Interview 2: Experiences – Healthcare Professionals**

**Initial thoughts**

- What are your initial reflections on how the transition to Remote Patient Monitoring (RPM) has progressed?
- In your view, which factors were most critical for a successful implementation?
- To what extent did the training and information you received in advance prepare you to carry out RPM?
- How did you experience the support provided by the supplier, management, or designated supervisors when encountering challenges?

**Experiences with patient monitoring**

- How would you describe the patient monitoring process through RPM compared to previous routines?
- Can you elaborate on any advantages you have experienced following the implementation of RPM?
- Prior to implementation, concerns were raised about the potential loss of important clinical cues—for instance, that patients might underreport adverse effects in digital forms, or that reduced face-to-face interaction could lead to missed symptoms. Do you have any examples illustrating this?
- In your perception, how have patients responded to digital follow-up?
- Have you received any positive feedback from patients? Please provide examples.
- Have you received any negative feedback? Please provide examples.
- To what extent have your initial concerns been confirmed or disproved during practice?
- Has patient involvement or participation in care been affected by the introduction of RPM? If so, how?

**Experiences with work practice and professional role**

- In what ways has RPM influenced your everyday work and professional responsibilities?
- What positive changes have you experienced?
- What challenges or negative consequences have arisen?
  - If so, in what ways?
- Has RPM contributed to more efficient work processes?
  - If not, why not?
- In earlier interviews, some healthcare professionals expressed concern that RPM could lead to nurses becoming overworked by increasing interactions with only the most complex patients. What is your view on this?
- Have you encountered any specific challenges along the way, and how have you dealt with them?
- Overall, do you consider RPM to be a useful tool?
- In what way, patients, professionals, management, or the healthcare system?
- Do you think RPM should be established as a permanent service offering?
  - Why or why not?
